# Reliable Transmission Optimization for UAV-Relayed Space–Air–Ground Integrated Vehicular Networks

**DOI:** 10.3390/s26165279

**Published:** 2026-08-20

**Authors:** Liang Zong, Yun Cheng, Yi Yao

**Affiliations:** 1 College of Information, Hunan University of Humanities, Science and Technology, Loudi 417000, China; zongliang@huhst.edu.cn (L.Z.); yuncheng@huhst.edu.cn (Y.C.); 2 College of Information Science and Engineering, Shaoyang University, Shaoyang 422000, China

**Keywords:** space–air–ground integrated networks (SAGINs), unmanned aerial vehicle (UAV), vehicular networks, reliable transmission

## Abstract

Driven by the vision of sixth-generation (6G) communication networks, Space–Air–Ground Integrated Vehicular Networks (SAGVNs) address the connectivity blind spots inherent in traditional networks by integrating unmanned aerial vehicles (UAVs) as highly mobile relay nodes. However, the high bit error rates (BERs) and prolonged propagation delays characteristic of satellite links, coupled with the highly dynamic topologies and multi-hop transmission nature of UAVs and terrestrial vehicles, present significant challenges to reliable end-to-end data streaming. To mitigate the performance degradation caused by link asymmetries in heterogeneous networks, this paper proposes a reliable transmission optimization scheme for UAV-relayed SAGVNs. By comprehensively modeling the transmission dynamics of long-delay, high-BER satellite links and mobile multi-hop UAV networks, the proposed scheme introduces an enhanced slow-start mechanism to accelerate throughput growth, thereby mitigating the startup lag induced by extensive propagation delays. Furthermore, an accurate packet loss differentiation model is established during the congestion avoidance phase. This model effectively decouples non-congestion packet losses—triggered by random channel errors or topology handovers due to high-speed node mobility—from genuine congestion-induced losses caused by buffer overflows at bottleneck nodes. Simulation results demonstrate that the proposed adaptive scheme demonstrates notable improvements over classical loss-based and delay-based baselines in reducing queuing delays at UAV relay nodes, enhances the transmission efficiency of multi-hop terminals, and effectively maintains end-to-end goodput stability in high-latency environments.

## 1. Introduction

Driven by the architectural evolution toward sixth-generation (6G) mobile communication networks, the establishment of a novel communication paradigm characterized by full-space coverage and ubiquitous connectivity has become widely recognized in both academia and industry. Against this backdrop, the deep integration of Space–Air–Ground Integrated Networks (SAGINs) and terrestrial Vehicular-to-Everything (V2X) networks has yielded a highly promising three-dimensional network topology. Within this architecture, unmanned aerial vehicles (UAVs) are rapidly evolving from dedicated reconnaissance and surveillance tools into pivotal aerial nodes. Owing to their flexible 3D deployment, high probability of line-of-sight (LoS) transmission, and superior mobility, UAVs are now capable of providing emergency communication relaying and wide-area data coordination. This architecture effectively bridges the coverage gaps of traditional networks in scenarios involving damaged infrastructure or blind spots, achieving dynamic coordination and the complementarity of space, air, and ground resources. However, ensuring efficient and reliable end-to-end data stream transmission across such a highly heterogeneous network architecture remains a critical challenge in current protocol stack design.

Seamlessly embedding UAV relay networks into the space–air–ground architecture to serve mobile terminals presents significant cross-layer challenges spanning both the physical and network layers [1,2,3]. Unlike terrestrial networks governed by relatively stable parameters, space–air–ground–vehicle heterogeneous links exhibit profound asymmetries across both spatial and temporal scales. Specifically, satellite links are inherently constrained by excessive propagation delays due to vast physical distances, coupled with high bit error rates (BERs) over complex satellite-to-ground channels. Concurrently, the low-altitude UAV networks tasked with relaying operate under highly dynamic mobility, driving high-frequency network topology reconfigurations and severe multi-hop relay instability. This highly dynamic topology not only exacerbates the risk of routing disruptions but also induces pronounced fluctuations in end-to-end round-trip times (RTTs) and available bandwidth capacity.

Confronted with these complex hybrid network scenarios, existing end-to-end transport control protocols (e.g., TCP Reno, TCP Cubic) exhibit significant adaptability limitations. The fundamental algorithmic bottleneck stems from the fact that conventional TCP congestion control mechanisms rely on the rigid, implicit assumption that “packet loss implies congestion,” thereby lacking the fine-grained capability to discern the root causes of packet loss. In heterogeneous space–air–ground–vehicle links, packet losses observed at the receiver are frequently triggered by topology handovers resulting from high-speed UAV mobility, deep channel fading, or random bit errors on satellite links [4,5,6]. However, standard sender-side algorithms cannot accurately differentiate these non-congestion-related link anomalies from genuine network congestion caused by buffer overflows at bottleneck routers.

This absence of an accurate packet loss attribution mechanism leads to severe performance degradation. When the system encounters non-congestion packet loss, traditional transport-layer protocols erroneously trigger congestion avoidance mechanisms, aggressively shrinking the congestion window and causing a precipitous drop in the transmission rate. In long-delay hybrid environments, the recovery phase following a window reduction is exceedingly slow, leaving valuable spectrum resources underutilized for extended periods and precipitating a severe collapse in throughput. Furthermore, topology reconfigurations driven by high-frequency UAV mobility often cause transient routing disruptions. If the transport-layer protocol fails to rapidly adapt to these intermittent connectivity characteristics, end-to-end Quality of Service (QoS) fluctuations will intensify, potentially culminating in connection dropouts.

Although extensive research has been conducted in recent years regarding physical-layer waveform design, MAC-layer scheduling, and network-layer routing optimization [7,8,9,10], adaptive end-to-end congestion control mechanisms tailored specifically for heterogeneous integrated scenarios remain scarce. Existing optimization schemes primarily focus on local optimizations within a single domain (e.g., pure satellite or pure ad hoc networks), limiting their direct applicability to cross-domain space–air–ground integrated environments. Alternatively, they rely on complex cross-layer signaling interactions, which introduce prohibitive overhead and latency in highly dynamic networks.

Motivated by these challenges, this paper aims to address the limitations of traditional transport control by proposing an adaptive end-to-end congestion control scheme tailored for heterogeneous environments. Grounded in the coupled transmission characteristics of long-delay, high-BER satellite links and highly dynamic multi-hop UAV networks, this study seeks to mitigate the “spurious congestion” dilemma inherent in integrated heterogeneous networks. The main innovations and contributions of this paper are summarized as follows:

Enhanced Slow-Start Mechanism: By estimating available bandwidth, the proposed scheme attempts to probe link capacity during the initialization phase to improve data throughput, thereby alleviating the startup lag induced by extensive propagation delays.
Packet Loss Differentiation Model: Utilizing real-time link status metrics, such as RTT and corresponding delay intervals, the model aims to identify the root causes of packet loss. This algorithm is designed to reduce unnecessary rate back-offs during non-congestion scenarios, such as channel fading or topology handovers.
Dynamic Congestion Avoidance Strategy: The congestion window evolution law is adaptively adjusted based on the identified packet loss classification. To better capture the actual processing efficiency of the underlying protocol stack in multi-hop heterogeneous networks, bits per second (bps) is adopted as a key evaluation metric for throughput.

## 2. Related Work

### 2.1. Transmission in Heterogeneous SAGIN and Vehicular Networks

Within the heterogeneous integration of space–air–ground integrated networks (SAGINs) and vehicular networks, architectural design and resource scheduling constitute the dual pillars for enabling reliable transmission. At the macro-architectural design level, Hui et al. [11] leveraged digital twin (DT) technology to establish an intelligent space–air–ground–vehicle integrated network (ISAGIVN) framework, providing an in-depth analysis of virtualized deployment, state synchronization, and dynamic migration mechanisms. Driven by the rapid development of Low Earth Orbit (LEO) satellite networks and SAGINs, transmission optimization tailored for dynamic heterogeneous network environments has garnered widespread attention. To mitigate TCP performance degradation induced by time-varying links, satellite handovers, and dynamic routing updates in LEO satellite networks, Cao et al. [12] proposed SaTCP-LEO, a cross-layer optimization mechanism. By leveraging the predictability of satellite orbits to assist TCP congestion control decisions, this mechanism significantly enhances network throughput while ensuring fairness. However, this approach is primarily designed for the dynamics of LEO satellite links and does not adequately account for the complex topological changes and random link perturbations introduced by Unmanned Aerial Vehicle (UAV) relays and terrestrial multi-hop networks. Furthermore, Zhang et al. [13] systematically reviewed the key technologies and challenges in UAV-assisted TCP-SAGIN, highlighting that dynamic topology management, cross-layer protocol adaptation, resource optimization, and reliable transmission control remain critical bottlenecks hindering the practical deployment of SAGINs. Therefore, achieving highly reliable and efficient transmission control under the coupled effects of multiple dynamic factors—such as prolonged satellite propagation delays, high-speed UAV mobility, and terrestrial multi-hop transmissions—remains an open challenge requiring further investigation. Balancing transmission rates against energy efficiency, Xu et al. [14] formulated an energy-efficient joint resource allocation (ERDM) framework based on multi-agent deep reinforcement learning, achieving global optimization for both UAV trajectories and transmit power allocation.

Regarding physical-layer channel optimization, Liu et al. [15] integrated simultaneously transmitting and reflecting reconfigurable intelligent surfaces (STAR-RIS) into high-speed railway communications, maximizing the aggregate transmitted bits over selected links via a coalitional game and QoS-aware scheduling. For the granular orchestration of heterogeneous computational tasks, Abbas et al. [16] devised a partial offloading policy leveraging the twin delayed deep deterministic (TD3) policy gradient algorithm to enable efficient collaborative offloading by routing delay-sensitive tasks to UAVs and compute-heavy tasks to LEO satellites. Additionally, to accommodate diverse vehicular service priorities, Pandey et al. [17] presented a novel Q-learning-based resource allocation model that upholds strict QoS guarantees for mission-critical applications, such as autonomous driving, even amidst surging network demands. Motivated by the ubiquitous connectivity imperatives of the emerging low-altitude economy, Zhou et al. [18] constructed a space–air–ground low-altitude aircraft vehicular network (SAG-LAAVN) featuring a hyper-converged architecture, validating the seamless coverage efficacy of a fully decoupled network paradigm within three-dimensional intelligent transportation systems. Finally, to alleviate device-level resource bottlenecks while safeguarding data privacy in heterogeneous settings, Li et al. [19] proposed a collaborative federated learning framework over UAV-assisted device-to-device (D2D) communications, employing an iterative resource allocation (IRA) algorithm to minimize global energy consumption and accelerate training convergence.

### 2.2. Transmission Control in UAV and Vehicular Networks

Within UAV-assisted vehicular ad hoc networks, high topological dynamics and channel heterogeneity place significant demands on underlying transport-layer control, routing decisions, and collaborative orchestration. Focusing on congestion control and emergency message dissemination, Hemmati et al. [20] developed a traffic-aware UAV-assisted fog computing congestion control strategy (UFC3) tailored for 5G-enabled vehicular emergency communications, ensuring resilient connectivity between distressed vehicles and remote servers. To alleviate backhaul congestion and enhance power utilization efficiency, Wang et al. [21] derived a closed-form expression for the UAV offloading rate and devised an alternating optimization algorithm coupling cooperative caching with power control to maximize system-wide energy efficiency. At the network layer, addressing node selfishness in real-world deployments, Ye et al. [22] designed an integrated opportunistic forwarding scheme that evaluates node centrality alongside buffer status, substantially reducing data delivery delays.

Driven by the pervasive integration of intelligent paradigms, Ali et al. [23] systematically surveyed state-of-the-art artificial intelligence and machine learning (AI/ML) applications in UAV vehicular network resource and trajectory management, detailing the critical trade-offs among computational overhead, empirical data acquisition, and model complexity. To guarantee seamless real-time coordination among multi-UAV swarms, Savkin et al. [24] fused dynamic programming with model predictive control to formulate a joint downlink transmission scheduling and trajectory planning policy that adheres to rigorous collision-avoidance constraints, providing formal mathematical proofs of its optimality. Moving beyond the scalability and trust vulnerabilities of centralized paradigms, Alagha et al. [25] developed a transparent coordination framework integrating blockchain with multi-agent deep reinforcement learning (MDRL), utilizing a bilateral preference mechanism to enable decentralized relay selection and persistent coverage maintenance. For dynamic edge routing optimization, Zhang et al. [26] developed a geographic edge routing protocol assisted by a double deep Q-network (DDQN), empowering vehicles to autonomously learn environmental dynamics and utilize an experience replay buffer to select the optimal next hop, thereby enhancing the packet delivery ratio (PDR). Finally, to minimize the overall operational costs of UAV-enabled mobile edge computing (MEC) systems, Fang et al. [27] proposed a distributed optimization scheme via the alternating direction method of multipliers (ADMM), tractably reformulating the underlying non-convex problem into convex subproblems to significantly accelerate convergence and maximize global system utility.

The remainder of this paper is organized as follows: Section 3 analyzes the UAV-relayed space–air–ground integrated vehicular network model; Section 4 presents a reliable transmission scheme for the proposed network model; Section 5 evaluates the proposed scheme; and Section 6 concludes the paper.

## 3. UAV-Relayed Space–Air–Ground Integrated Vehicular Network

### 3.1. Architecture Model: Multi-Dimensional Three-Dimensional Heterogeneous Coordination Mechanism

**Figure 1:**
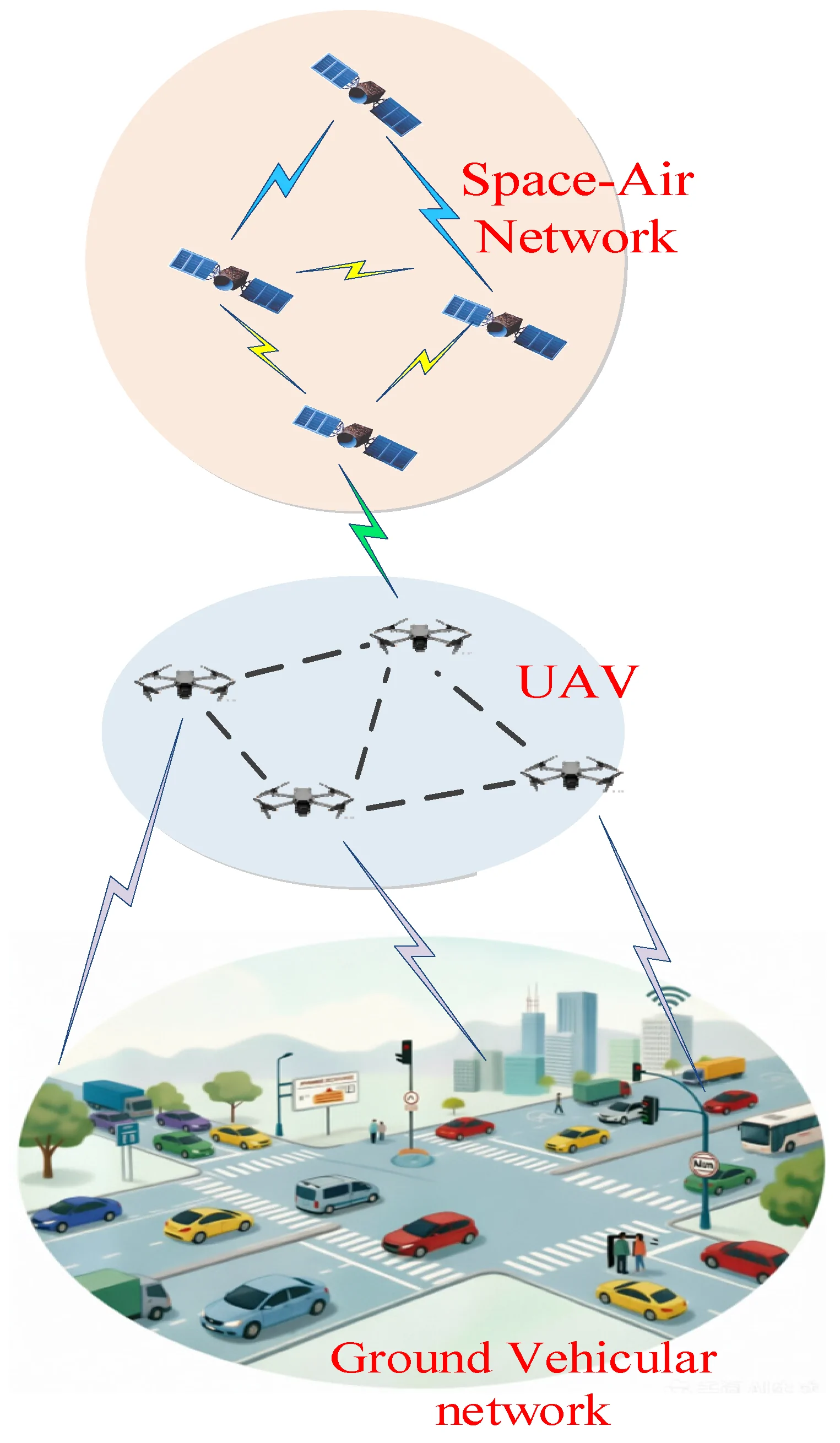
UAV-relayed Space–Air–Ground integrated vehicular network.

In high-reliability Vehicle-to-Everything (V2X) application scenarios, such as future smart cities, highway emergency communications, and autonomous driving platoons, standalone terrestrial communication infrastructure struggles to meet the stringent demands for ubiquitous and continuous coverage. To address these limitations, this paper develops a space–air–ground integrated multi-hop UAV network model tailored for vehicular networks. As illustrated in Figure 1, the Space–Air–Ground Integrated Network (SAGIN) establishes a multi-layered, three-dimensional communication architecture by integrating Geosynchronous Earth Orbit (GEO) satellites, Low Earth Orbit (LEO) satellites, Unmanned Aerial Vehicle (UAV) networks, and terrestrial networks.

Within this proposed architecture, the network is vertically partitioned into four functional layers, enabling multidimensional resource mapping via cross-layer interfaces:

Top-Tier GEO Satellite Layer: This layer provides wide-area coverage and backbone backhaul. Although constrained by significant propagation delays, GEO satellites offer extensive beam footprints that form the space-based backbone. They provide macro-connectivity to remote blind spots inaccessible by terrestrial infrastructure and to disaster areas with compromised facilities.

Mid-Tier LEO Satellite Layer: Characterized by lower latency attributes compared to GEO systems, this layer is responsible for regional relaying. LEO constellations form dynamic topologies via inter-satellite links (ISLs), thereby substantially reducing the end-to-end propagation delay.

Lower UAV Network Layer: This layer operates as aerial mobile base stations or relay nodes, offering significant deployment flexibility. In vehicular network scenarios, UAV swarms constitute an aerial wireless mesh network. These UAVs can operate as high-altitude platforms (HAPs) or low-altitude platforms (LAPs) to interface directly with satellite links, or act as aerial relays to dynamically connect with terrestrial vehicular users.

Bottom-Tier Terrestrial Network: This layer comprises urban infrastructure, vehicles, and user terminals. Due to the high mobility of terrestrial equipment—such as autonomous vehicles, on-board units (OBUs), roadside units (RSUs), and Internet of Things (IoT) sensors—their uplink and downlink connections frequently undergo handovers among terrestrial base stations, UAV nodes, and satellite links.

While this three-dimensional architecture significantly expands network coverage and enhances topological robustness, it simultaneously introduces substantial challenges in channel modeling and transport-layer protocol design for cross-domain heterogeneous networks.

### 3.2. High Dynamic Link Evolution and Vehicular Channel Characteristics

Within this highly heterogeneous network, communication links exhibit pronounced dynamics. Compared to conventional static networks, the space–air–ground–vehicle integrated network presents complex channel characteristics during spatial propagation, characterized by high mobility, susceptibility to intermittent disruptions, and significant latency variations.

Specifically, the multi-hop links within the UAV layer, as well as the links between the UAV layer and the terrestrial vehicular nodes, are highly susceptible to blockage by obstacles in the three-dimensional environment, such as high-rise buildings and dense foliage. When a UAV rapidly traverses behind a building, or when a terrestrial vehicle enters a signal shadow region like a tunnel or an urban canyon, the line-of-sight (LoS) link can be instantaneously severed, resulting in bursty and extensive packet losses at the lower layers. Concurrently, the high-speed mobility of UAVs in 3D space, coupled with the relative motion of terrestrial vehicles (particularly vehicular platoons on highways), induces severe Doppler shifts, which further degrades physical-layer demodulation performance.

Beyond the challenges of spatial blockage and relative motion, the introduction of heterogeneous relay links substantially alters the end-to-end latency distribution profile. The prolonged propagation delays and periodic handovers inherent to satellite links further exacerbate the instability of the end-to-end path. Under multi-hop relay routing mechanisms, the end-to-end round-trip time (RTT) can exhibit severe fluctuations, ranging from the millisecond scale (e.g., via direct terrestrial base stations or UAV-to-vehicle links) to hundreds of milliseconds (e.g., when data is routed through GEO/LEO satellites). This pronounced RTT jitter, triggered by instantaneous topological reconfigurations, renders traditional transport protocols—which are typically predicated on fixed assumptions or stationary stochastic processes—unable to sustain highly efficient data delivery.

### 3.3. Failure Analysis and Theoretical Bottlenecks of Traditional End-to-End Congestion Control Mechanisms

In the aforementioned space–air–ground–vehicle network architecture, the primary challenge for the transport layer is ensuring reliable transmission over highly uncertain channels. Transport protocols must be capable of differentiating between physical-layer link disruptions and genuine, load-driven network congestion, while maintaining stability across paths with heterogeneous latency profiles. However, legacy TCP—the de facto standard for internet transport—bases its congestion control logic on the rigid “loss-indicates-congestion” paradigm, an assumption that proves fundamentally inadequate in SAGIN-enabled UAV networks.

When optimizing reliable transmission for UAV-relayed SAGVNs, conventional transport protocols rely predominantly on packet loss as the primary indicator of network congestion, a mechanism that suffers from significant ambiguity in highly dynamic topologies. The underlying logic implicitly equates packet drops with buffer overflows at bottleneck nodes, subsequently triggering aggressive reductions in the transmission rate. However, in UAV-assisted space–air–ground networks, transient packet losses induced by environmental factors—such as physical blockages, multipath fading, and intermittent link disconnections—are commonplace. Adhering to this legacy logic misinterprets channel-induced losses as network congestion, triggering spurious congestion avoidance and leading to the unnecessary collapse of the end-to-end congestion window. Particularly in satellite-relayed links characterized by large bandwidth-delay products (BDPs), this misclassification prevents the data stream from rapidly recovering link utilization. Consequently, expensive space–ground spectrum resources remain underutilized for extended periods, severely degrading the throughput performance of high-traffic vehicular services.

Concurrently, delay-based congestion control algorithms that monitor real-time RTT fluctuations exhibit a fundamental incompatibility with the UAV-relayed SAGIN-V2X architecture. The core assumption of these approaches’ attributes RTT increments solely to queuing delays at bottleneck routers. In SAGINs, however, pronounced RTT jitter is primarily driven by physical propagation delay variations stemming from satellite orbital mechanics, the highly mobile trajectories of UAV relays, and the frequent handovers of terrestrial vehicles. During dynamic multi-hop routing reconfigurations, RTT fluctuations induced by geometric topological evolution are often on the same order of magnitude as queuing delays caused by traffic surges. Because legacy protocols fail to accurately decouple physical-layer topological variations from transport-layer congestion, their high sensitivity to delay fluctuations precipitates severe oscillations in the transmission rate, ultimately undermining the continuity and stability required for vehicular communications.

Furthermore, when evaluating reliable transmission performance in such highly dynamic networks, conventional throughput metrics exhibit notable limitations. Relying solely on bits per second (bps) as a macroscopic indicator often obscures the impact of transient, bursty packet losses on real-time vehicular control signaling. Consequently, reliability analysis at the vehicular control plane must incorporate finer-grained metrics—such as the packet processing rate measured in packets per second (pps)—to accurately capture the responsiveness of congestion control algorithms under rapid topological shifts. Because conventional TCP variants lack cross-layer topology awareness, they can neither exploit the redundant paths of GEO/LEO satellites for rapid failover nor seamlessly adapt to the frequent reconfigurations inherent in UAV networks. Therefore, engineering an adaptive, end-to-end congestion control mechanism capable of sensing SAGIN states and accurately decoupling link anomalies from genuine network congestion is essential for optimizing the transmission performance of UAV-assisted vehicular networks.

## 4. Reliable Transport Control Algorithm for UAV-Relayed Space–Air–Ground Integrated Vehicular Networks

In the Space–Air–Ground Integrated Vehicular Network (SAGIN), unmanned aerial vehicle (UAV) relay links frequently exhibit high propagation delays, severe topological dynamics, and complex channel fading characteristics. Traditional end-to-end Transmission Control Protocols (TCP) rely on rudimentary packet-loss-driven or fixed delay-gradient mechanisms, rendering them inadequate for effectively differentiating between congestion-induced packet loss and random channel errors in such heterogeneous environments. Furthermore, excessive Round-Trip Times (RTT) precipitate a drastic degradation in the convergence speed of conventional algorithms during the bandwidth probing phase. To circumvent this transmission bottleneck, this section proposes a novel adaptive end-to-end congestion control mechanism tailored for heterogeneous networks. By formulating a virtual queue backlog estimator alongside a spatio-temporally normalized ordinary differential equation (ODE) model, the proposed mechanism guarantees topological fairness in the throughput dimension while simultaneously achieving random error immunity and delay decoupling.

### 4.1. Virtual Queue Backlog Estimation and Bottleneck State Awareness

Within heterogeneous network topologies incorporating UAV relays, the queuing depth of packets at bottleneck link routers or relay node buffers serves as a core parameter indicating whether the network has transitioned into a substantive congestion state. Consequently, this study establishes a queue model based on fluid-flow approximation, introducing a virtual queue backlog state variable, Q(t), to achieve continuous-time estimation of the end-to-end link queuing state.

Let $\tau_{i}$ denote the instantaneous round-trip delay of the i-th packet sampled at the sender. Let $\tau_{min}$ denote the minimum round-trip delay observed since the current connection was established, a parameter that physically approximates the sum of the base propagation delay and the static processing delay over the end-to-end link.

At time t, assuming the current congestion window size is W(t), the instantaneous actual throughput $\lambda_{act}(t)$ (in pps) injected into the network can be expressed according to closed-loop queuing network theory as:

(1) $$\lambda_{act}(t)=\frac{W(t)}{\tau_{i}}$$

To adapt to sudden route changes and topology switches, $\tau_{min}$ is continuously updated using a sliding time-window rather than a static global minimum, ensuring the packet loss classifier remains accurate post-handover. Assuming the network is currently in an absolutely ideal, unloaded state—meaning no packets are queued at the bottleneck node ($\tau_{i}\to \tau_{min}$)—the theoretical maximum expected throughput $\lambda_{exp}(t)$ attainable by the system is defined as:

(2) $$\lambda_{exp}(t)=\frac{W(t)}{\tau_{min}}$$

Based on the fluid throughput differential, the virtual queue backlog Q(t) currently residing within the bottleneck node’s buffer can be derived. In transient scenarios such as sudden topology handovers where the bottleneck queue clears instantaneously, Q(t) mathematically resets to near zero, seamlessly triggering the Slow-Start probing mode. At any observation instant t, Q(t) is equivalent to the integral of the difference between the expected throughput and the actual throughput over the timescale of the base propagation delay, formulated as:

(3) $$Q(t)=(\lambda_{exp}(t)-\lambda_{act}(t))\times \tau_{min}$$

Combining the aforementioned system of equations yields the discretized, dimensionless expression for the virtual queue backlog:

(4) $$Q(t)=W(t)\cdot \left(1-\frac{\tau_{min}}{\tau_{i}}\right)=W(t)\frac{\tau_{i}-\tau_{min}}{\tau_{i}}$$

This formula indicates that the variable Q(t) synthesizes the current volume of injected data with the evolutionary trend of the link’s delay gradient, functioning as a predictive indicator for link state warnings. To enable discrete state transitions, a state decision threshold θ is defined. Grounded in system stability margin analysis, an empirical value of θ=3 effectively filters out minor delay jitter induced by underlying MAC protocol scheduling. Hence, the congestion state indicator function $I_{c}(t)$ is defined as follows:

- When Q(t)<θ, $I_{c}(t)=0$, indicating that the link is in a “non-congested state” and network bandwidth redundancy remains.
- When Q(t)≥θ, $I_{c}(t)=1$, indicating a massive packet backlog in the buffer, signifying that the link has entered a “congested state”.

### 4.2. Continuous-Time Spatiotemporal Scale Normalization Model

Traditional Additive Increase Multiplicative Decrease (AIMD) mechanisms rely heavily on clocking driven by acknowledgment characters (ACKs). This implicit evolutionary mechanism causes UAV or satellite relay links with long RTTs to suffer severe performance degradation when competing for bandwidth against terrestrial links with short RTTs. To eradicate the fairness deviation caused by RTT heterogeneity, this study introduces a spatio-temporal scale normalization mechanism, forcing the derivative of the system’s instantaneous transmission rate to be independent of the specific delay characteristics of the underlying physical links.

Without loss of generality, a system reference round-trip delay $\tau_{ref}$ is defined. In practical parameter mapping, $\tau_{ref}$ typically adopts the average propagation delay of typical terrestrial wired networks (e.g., set to 25 ms, $\tau_{ref}=25$ ms is empirically selected aligning with standard terrestrial wired network propagation, serving as an optimal normalization baseline). Accordingly, a dimensionless spatio-temporal-scale scaling factor κ is constructed:

(5) $$\kappa =\frac{\tau_{i}}{\tau_{ref}}$$

To prevent instantaneous buffer overflow induced by extreme propagation delays (e.g., GEO links), an upper bound $\kappa_{max}$ is enforced. Based on the bottleneck buffer capacity, $\kappa =min(\tau_{i}/\tau_{ref},\kappa_{max})$. The core mathematical constraint of this scaling factor lies in reconstructing the ODE for congestion window growth in the continuous-time domain. In standard TCP evolution models, the growth rate during the congestion avoidance phase can be approximated as $\frac{dW(t)}{dt}=\frac{1}{\tau_{i}}$. To strictly equalize the growth rate of long-delay connections per unit time with that of the baseline connection (i.e., a connection where $\tau_{i}=\tau_{ref}$), the growth derivative of the target window $W^{*}(t)$ must be aligned and calibrated. The calibration condition for the differential equation is:

(6) $$\frac{dW^{*}(t)}{dt}=\frac{\kappa }{\tau_{ref}}$$

The factor κ equivalently maps the discrete increment function—which traditionally depends on heterogeneous physical delays—into a globally normalized evolution equation, providing a theoretically bounded growth baseline for subsequent discrete-time nonlinear window updates.

### 4.3. Queue-State-Driven Nonlinear Window Evolution Control Law

Based on the previously derived virtual queue state parameter *Q*(*t*) and the scale scaling factor κ, this subsection further designs the dynamic control laws for the Slow Start and Congestion Avoidance phases in discrete states. To suppress micro-bursts induced by large window step changes and the consequent buffer underflow/overflow, the sender’s lower layers strictly couple with a packet spacing mechanism to ensure smooth transmission.

#### 4.3.1. Adaptive Scaled Slow-Start Model

During the initial slow-start phase of connection establishment, traditional mechanisms exhibit a total window increase of one Maximum Segment Size (MSS) for every non-duplicate ACK received. To compensate for the lag effect of long RTTs during the exponential growth phase, the scaling factor κ is introduced for nonlinear compensation. When the system receives the n-th new ACK, the updated congestion window $W_{n+1}^{*}$ evolves according to the following difference equation:

(7) $$W_{n+1}^{*}=W_{n}^{*}+2^{\kappa -1}$$

From a dynamical systems perspective, this equation expands the single-step constant increment into a nonlinear term exponentiated by κ. Thus, it can be proven that regardless of the scale of the $\tau_{i}$ offset introduced by the 3D spatial positioning of UAV nodes or the propagation delays of satellite orbits, this mechanism ensures that the instantaneous transmission rate (pps) of the end-to-end connection over the time domain t converges to the theoretical value of the low-latency reference link.

#### 4.3.2. Queue-Aware Congestion Avoidance Model

Once $W^{*}(t)$ surpasses the slow-start threshold, the algorithm transitions into the congestion avoidance phase. At this stage, the control system algebraically fuses the queuing state parameter Q(t) with the scale compensation parameter κ to formulate a multi-stable piecewise additive increase control law:

- State A (active bandwidth probing: Q(t)<θ): This state indicates that available bandwidth capacity persists in the network, and the bottleneck queue has yet to experience substantive backlog. The control law aims to aggressively probe bandwidth utilizing the scale compensation mechanism. Theoretically, over a complete RTT cycle, the total target window increment should be scaled to $\kappa^{2}$. Discretizing this increment and allocating it to individual ACK reception events yields the following difference update equation:

(8) $$W_{n+1}^{*}=W_{n}^{*}+\frac{\kappa^{2}}{W_{n}^{*}}$$

- State B (congestion evasion and steady-state residence: Q(t)≥θ): When Q(t) breaches the critical threshold θ, it signals that the bottleneck link bandwidth is approaching saturation. Sustaining the original growth derivative would highly likely precipitate bursty buffer overflows. Accordingly, a delayed evolution mechanism is designed: by artificially suppressing the first derivative of the congestion window’s growth, the system is forced to “reside” near the extreme operational point of high throughput. Mathematically, the single-step update stride is attenuated to half of the standard compensation amount:

(9) $$W_{n+1}^{*}=W_{n}^{*}+\frac{\kappa^{2}}{2W_{n}^{*}}$$

In the specific engineering implementation within the protocol stack, this equation is equivalent to introducing an acknowledgment counter, executing an incremental update operation of magnitude $\frac{\kappa^{2}}{W_{n}^{*}}$ only after two valid new ACKs are received consecutively. By dampening the controller’s sensitivity, this mechanism effectively diminishes the probability of self-induced congestion packet loss.

### 4.4. Error-Congestion Orthogonal Decoupling and Multi-Dimensional Multiplicative Decrease Strategy

Within the highly complex SAGIN channel environment, UAV relay links are exceptionally susceptible to multipath fading, Doppler shifts, and environmental noise, engendering high-frequency random bit errors. Conventional algorithms uniformly treat such packet losses as congestion signals, thereby triggering blind multiplicative decreases that cause severe deterioration in throughput performance. Upon triggering a fast retransmit (e.g., receiving 3 consecutive duplicate ACKs) or a Retransmission Timeout (RTO) event, the proposed algorithm invokes a state decoupling matrix to recalculate the slow-start threshold (ssthresh).

Let $t_{0}$ represent the exact moment the packet loss response mechanism is triggered. Introducing a window degradation penalty factor η, the algorithm executes a conditional hypothesis test based on the queue backlog state observation vector $Q(t_{0})$ at that moment:

**Hypothesis** **H0.** *(Random channel loss: *$Q(t_{0})<\theta$*): Because the virtual queue has not amassed a significant backlog, the system possesses sufficient confidence to determine that the current packet loss event is a random loss induced by physical layer channel noise on the UAV link. To avert a cliff-like collapse in the link transmission rate, the control law imposes only a mild penalizing feedback, setting the truncation factor to *η=0.8.

**Hypothesis** **H1.** *(Substantive congestion loss: *$Q(t_{0})\geq \theta$*): At this point, the queue backlog parameter has exceeded its limit, confirming that the packet loss event was precipitated by a buffer overflow at a network node. To guarantee the long-term stability and fair convergence of bandwidth allocation across the entire SAGIN topology, the control law applies stringent penalty measures, adhering to the classical AIMD paradigm by calibrating the truncation factor to *η=0.5.

The unified multiplicative decrease state equation can be formally expressed as:

(10) $$ssthresh=\lfloor \eta \times W^{*}(t_{0})\rfloor ,\;\mathrm{where}\;\eta =\left\{\begin{array}{lll} 0.8, & \mathrm{if}\;Q(t_{0})<\theta |0.5, & \mathrm{if}\;Q(t_{0})\geq \theta \end{array}\right.$$

Further analysis indicates that bursty interference within heterogeneous wireless channels frequently results in multiple consecutive packet losses within a single congestion window cycle. To intercept the “retransmission storms” triggered by repetitive back-offs over long-delay links, this mechanism strictly mandates support for the Selective Acknowledgment (SACK) option at the underlying receiver layer. By parsing SACK blocks, the sender can reliably recover all lost segments within a single RTT cycle, thereby sustaining the closed-loop stability of the control system.

The complete algorithm pseudocode is as follows (Algorithm 1):
**Algorithm 1:** Reliable Transport Control Algorithm for UAV-Relayed SAGVNs// Initialization τ_ref <- 25, θ <- 3            // τ_ref: System reference RTT; θ: Queue decision threshold τ_min <- ∞                                 // Minimum round-trip delay observed ack_counter <- 0                  // Counter for delayed evolution mechanism T_w <- 5.0                                // Sliding window size in seconds (Restored) t_last_update <- current_time()// Initialize time tracking (Restored) Procedure on_receive_new_ack(τ_i)     // Triggered on each new ACK |// 0. Sliding Time-Window Update for τ_min | if current_time()− t_last_update > T_w then | | τ_min <- τ_i | | t_last_update <- current_time() | else if τ_i < τ_min then | | τ_min <- τ_i | | t_last_update <- current_time() | end if | |// 1. Spatio-temporal Scale Normalization | κ_max <- 20.0                       // Empirically bounded by the maximum buffer capacity (Restored) | κ <- τ_i/τ_ref                  // Force derivative of transmission rate to be independent of delay | if κ > κ_max then | | κ <- κ_max | end if | |// 2. Virtual Queue Backlog Estimation | λ_act(t) <- W(t)/τ_i        // Instantaneous actual throughput (pps) | λ_exp(t) <- W(t)/τ_min      // Theoretical maximum expected throughput (pps) | Q(t) <- (λ_exp(t) −λ_act(t)) * τ_min// Continuous-time estimation of queuing state | |// 3. Bottleneck State Awareness | if Q(t) < θ then | | I_c(t) <- 0                   // Non-congested state, network bandwidth redundancy remains | else | | I_c(t) <- 1                   // Congested state, massive packet backlog in buffer | end if | |// 4. Queue-State-Driven Nonlinear Window Evolution | if W(t) < ssthresh then | | W(t) <- W(t) + 2^κ− 1      // Adaptive Scaled Slow-Start Phase | else | | if I_c(t) == 0 then          // State A: Active bandwidth probing | | | W(t) <- W(t) + κ^2^/W(t)// Base increment step per ACK | | | ack_counter <- 0 | | else                             // State B: Congestion evasion & steady-state | | | ack_counter <- ack_counter + 1// Delayed evolution mechanism to artificially suppress growth | | | if ack_counter >= 2 then | | | | W(t) <- W(t) + κ^2^/(2 * W(t))// Equivalent to half-increment overall | | | | ack_counter <- 0 | | | end if | | end if | end if Procedure on_packet_loss(t0)   // Triggered on Fast Retransmit (3 DUPACKs) or RTO | if Q(t0) < θ then              // Hypothesis H0: Random channel loss (e.g., channel noise) | | η <- 0.8                     // Mild penalizing feedback | else                               // Hypothesis H1: Substantive congestion loss (buffer overflow) | | η <- 0.5                         // Stringent penalty measures (AIMD paradigm) | end if | ssthresh <- ⌊η * W(t0)⌋       // Multiplicative decrease strategy | parse_SACK_blocks()            // Precise recovery of lost segments within a single RTT

## 5. Simulation Analysis

To comprehensively evaluate the proposed reliable transmission optimization mechanism for UAV-relayed Space–Air–Ground Integrated Vehicular Networks (SAGVNs), this study constructs a simulation platform cascading a space-based satellite network, an aerial unmanned aerial vehicle (UAV) relay layer, and a terrestrial vehicular network (V2X). The terrestrial network adopts a chain topology to establish a multi-hop wireless ad-hoc network, where receiving vehicular nodes are dynamically deployed from the first to the fourth hop, simulating end-to-end transmission characteristics across varying network edge coverage depths. The simulation platform OPNET 14.5 has link bandwidth 1.54 Mbps, buffer 16 Mbytes, packet size 1500 bytes. The >500 ms GEO RTT was implemented by explicitly configuring the satellite node’s propagation delay attribute in the OPNET topology to enforce a baseline one-way space-to-ground delay of 250 ms. The data traffic was generated using a continuous Constant Bit Rate (CBR) model (FTP/HTTP traffic models were also tested for validation). Each simulation scenario was executed for a duration of 600 s. To account for the stochastic nature of the mobility and channel error models, the reported results are averaged over 10 independent simulation runs using distinct random seeds to denote run-to-run variability. Before analyzing the core performance metrics, a brief sensitivity analysis was conducted to justify the empirical parameter selections. The reference delay $\tau_{ref}$ was set to 25 ms to emulate standard terrestrial baseline propagation. The queue decision threshold θ=3 was identified as the optimal inflection point to absorb routine MAC-layer medium access jitter without misclassifying it as congestion. Furthermore, the penalty factors 0.8 and 0.5 provide an orthogonal balance: 0.8 ensures rapid throughput recovery during transient channel noise, while 0.5 enforces strict AIMD fairness during genuine buffer saturation.

In this cascaded heterogeneous environment, the system simultaneously exhibits dual characteristics: macroscopic ultra-long propagation delays and microscopic highly dynamic channel degradations. The immense physical distance of the Geosynchronous Earth Orbit (GEO) satellite-to-ground link results in a round-trip time (RTT) typically exceeding 500 ms, inducing a severe “long fat pipe” effect characterized by a massive bandwidth-delay product (BDP). This substantial delay prevents the congestion window of traditional transport-layer protocols from reacting instantaneously to transient bottleneck states, easily triggering severe collapses in link utilization. Concurrently, satellite-ground channels inevitably suffer from spatial environmental disturbances such as atmospheric absorption, rain attenuation, and ionospheric scintillation, whereas low-altitude UAV relay and terrestrial V2X links are subjected to intense disruptions from high-speed three-dimensional node mobility, Doppler shifts, and instantaneous geometric topology reconfigurations. Under such extreme hybrid channels, even if the underlying physical and data link layers are equipped with advanced channel estimation and Forward Error Correction (FEC) coding, the transport-layer receiver will still experience prolonged, random, non-congestion bit errors, significantly degrading the convergence domain and accuracy of end-to-end congestion decisions.

To quantitatively reveal the adaptability of various congestion control laws to the aforementioned environment plagued by long delays and bursty errors, three representative classic mechanisms are selected as baselines for comparative analysis: Scheme 1 (TCP Reno: a classic loss-driven protocol [28]), Scheme 2 (TCP Veno: a protocol with wireless random loss discrimination [29]), and Scheme 3 (TCP Hybla: a protocol designed with RTT compensation for satellite links [30]). The background random bit error rate (BER) across the global wireless environment is uniformly configured at $1\times 10^{-5}$. The uniform BER of $1\times 10^{-5}$ represents the residual error rate post-Forward Error Correction (FEC) processing at the physical layer. For a standard Maximum Transmission Unit (MTU) frame size of 1500 bytes, this translates directly to the transport-layer Packet Error Rate (PER) evaluated in our simulations. The systematic evaluation focuses on three core metrics: link throughput rate, WLAN MAC layer medium access delay, and queuing delay at bottleneck intermediate nodes.

### 5.1. Analysis of Link Transmission Rate Mechanisms

**Figure 2:**
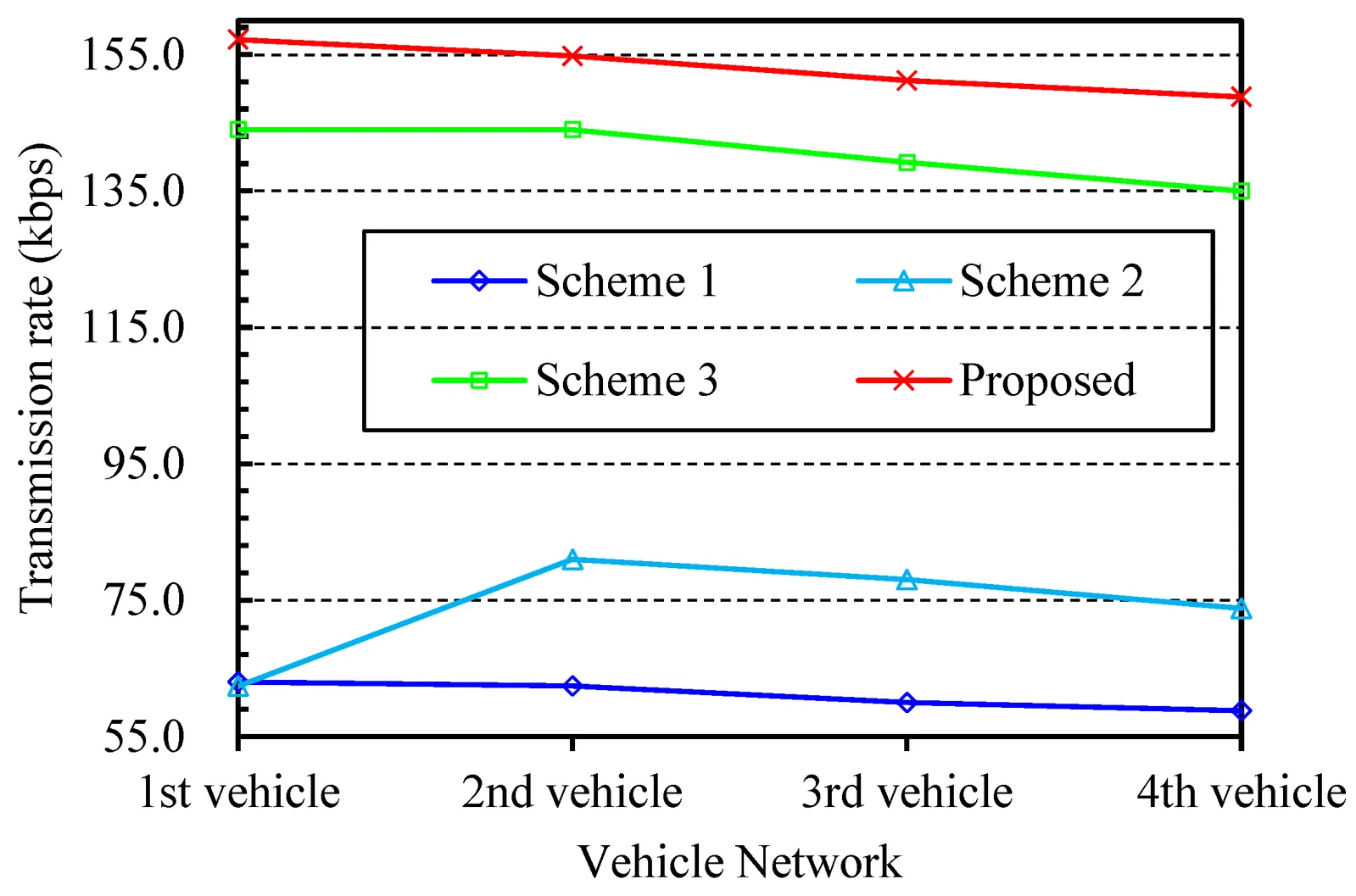
Transmission rate of the link in vehicle network.

Figure 2 illustrates the macroscopic link throughput rate trajectories under four groups of multi-hop vehicular node tests. The simulation curves demonstrate that the proposed adaptive scheme achieves optimal transmission rate performance across all test scenarios. Statistical data indicate that the average link transmission rate of the proposed scheme reaches 153.0 kbps, realizing a substantial performance gain of approximately 150.6% over classic Scheme 1 (averaging 61.05 kbps) and further unlocking about 8.86% more throughput potential compared to Scheme 3 (averaging 140.55 kbps). This explicit throughput gain directly validates the effectiveness of the proposed transport-layer state machine and time evolution equation designs, driven fundamentally by the following two core regulatory mechanisms.

Complete Elimination of Long Propagation Delay Lag via Spatiotemporal Scale Normalization: Traditional Scheme 1 exhibits severe performance degradation over long-delay satellite links, rooted in its discrete window evolution that adheres to the rigid, clock-driven constraint of increasing by one Maximum Segment Size (MSS) per RTT. In SAGVN scenarios where the RTT exceeds 500 ms and the BDP is massive, this sluggish step increase causes the congestion window (cwnd) growth to lag severely, leaving the valuable satellite spectrum pipe under-saturated and deadlocking throughput at a meager 60 kbps. In contrast, the proposed algorithm introduces a system reference round-trip delay $\tau_{ref}$ (calibrated to 25 ms) and constructs a dimensionless spatiotemporal scale scaling factor κ (Formula (5)). During the initial slow-start phase, the algorithm strictly decouples the growth derivative from the specific delay of the underlying physical link, compelling the updated congestion window to evolve exponentially according to a nonlinear difference equation (Formula (7)). Through this continuous-time derivative alignment calibration of the scaling factor (Formula (6)), the ability of the long-delay path to probe and occupy idle bandwidth per unit time receives strict equivalent compensation, effectively mitigating the inherent disadvantage of long-RTT connections when competing for cross-domain topological resources.

Immunity to Random Bit Errors via Error-Congestion Orthogonal Decoupling: Under a hostile channel environment with a BER of $10^{-5}$, the implicit assumption of “packet loss implies congestion” exposes severe adaptability flaws. Although Scheme 2 attempts to utilize discrete delay gradients to differentiate loss attributes, its lack of continuous-time fluid estimation for the bottleneck backlog depth makes it prone to attribution confusion under high-frequency wireless random bit errors, yielding a throughput (averaging 73.8 kbps) only marginally better than Scheme 1. The proposed algorithm constructs a virtual queue backlog estimator based on fluid-flow approximation, continually sampling the instantaneous RTT and the historically observed minimum delay to accurately extract the virtual backlog variable Q(t) residing in the bottleneck node’s buffer (Formula (4)). Upon triggering a packet loss response, the control system executes orthogonal decoupling based on conditional hypothesis testing (Formula (10)). If the virtual queue backlog has not breached the threshold ($H_{0}:Q(t)<\theta$), the state matrix determines the bursty loss is induced by physical-layer deep channel fading or UAV topology handovers, thus applying mild penalty feedback that safely locks the truncation factor at η=0.8. This mechanism effectively intercepts unnecessary window collapses caused by non-congestion link anomalies, ensuring the sender maintains a stable, high-order effective throughput exceeding 150 kbps. Compared to Scheme 3, the proposed scheme adopts a more proactive probing law when the network is unsaturated and controls the first derivative of the window to stably “reside” at the optimal operational extremum during the congestion avoidance phase (Formula (9)), successfully mining approximately 9% more network potential under heavy traffic loads.

### 5.2. WLAN MAC Delay Analysis for UAV Relay Nodes and Multi-Hop Vehicular Networks

From a statistical perspective, the WLAN MAC layer delay characterizes the actual medium access time required for a packet—after descending from the transport layer into the data link layer buffer—to successfully compete for and access the wireless physical channel. This metric is governed by the dual nonlinear coupling of the physical-layer wireless channel contention intensity and the traffic intensity injected downward by the transport layer.

**Figure 3:**
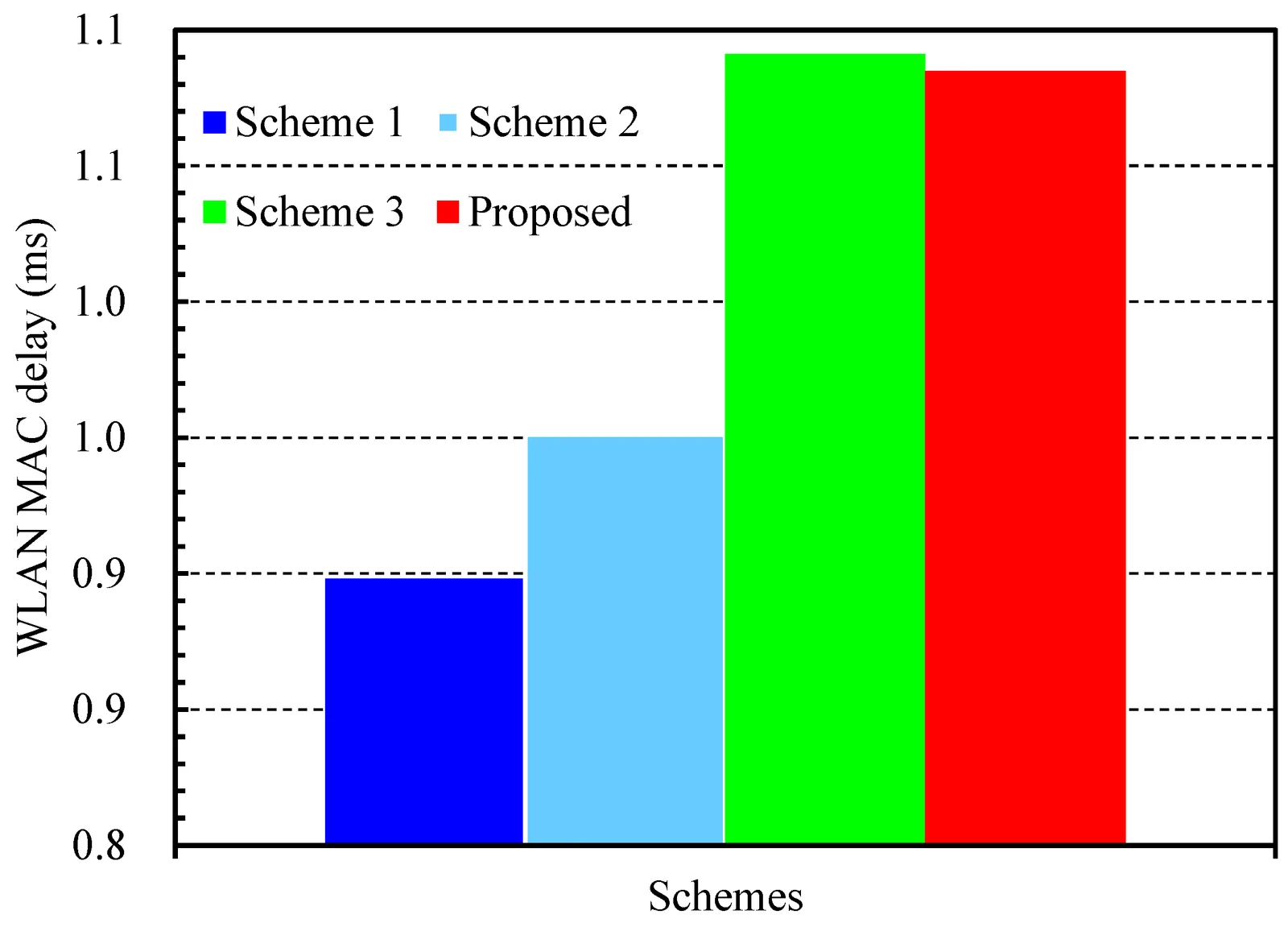
WLAN MAC delay for UAV relay.

According to the simulation output, intermediate UAV relay nodes exhibit a pronounced polarization effect in MAC layer delays induced by different congestion control algorithms under a hybrid network environment, as shown in Figure 3. Scheme 1 and Scheme 2 display relatively low MAC delays of 0.898 ms and 0.951 ms, respectively. This low-delay state is fundamentally an artifact of their conservative control laws; because they cannot identify physical-layer noise, random error events mis-trigger their multiplicative decrease mechanisms, causing the transmission window to severely shrink and rendering the traffic flow injected into the data link layer exceedingly sparse. This contention-free idle channel state, exchanged at the severe cost of end-to-end reliability, entirely deviates from the evolutionary demands for high-concurrency vehicular networks.

Conversely, operating on a long-delay expansion strategy, Scheme 3 blindly and aggressively inflates the transmission window due to its lack of decoupled awareness regarding underlying channel carrying capacity. This aggressive packet-injection strategy unloads an excessive volume of bursty data packets into the lower buffer within an extremely short period, triggering intense contention conflicts over the wireless channels between the UAV relay node and terrestrial multi-hop vehicular nodes. Fierce wireless medium collisions and frequently triggered binary exponential backoffs (BEB) morbidly inflate the packet medium access time, skyrocketing its MAC delay to 1.091 ms.

In contrast, the proposed optimization algorithm flattens the WLAN MAC layer delay to 1.085 ms while sustaining a high-throughput operation of 153.0 kbps, effectively lowering the delay overhead compared to the aggressive Scheme 3 mechanism. In a space–air–ground coupled architecture with a BER of $10^{-5}$, this reduction signifies that the mechanism effectively curbs the chaotic impact of micro-bursts on underlying wireless medium access by introducing implicit cross-layer packet spacing control at the transport layer, thereby alleviating channel congestion while adhering to the packet conservation principle.

**Figure 4:**
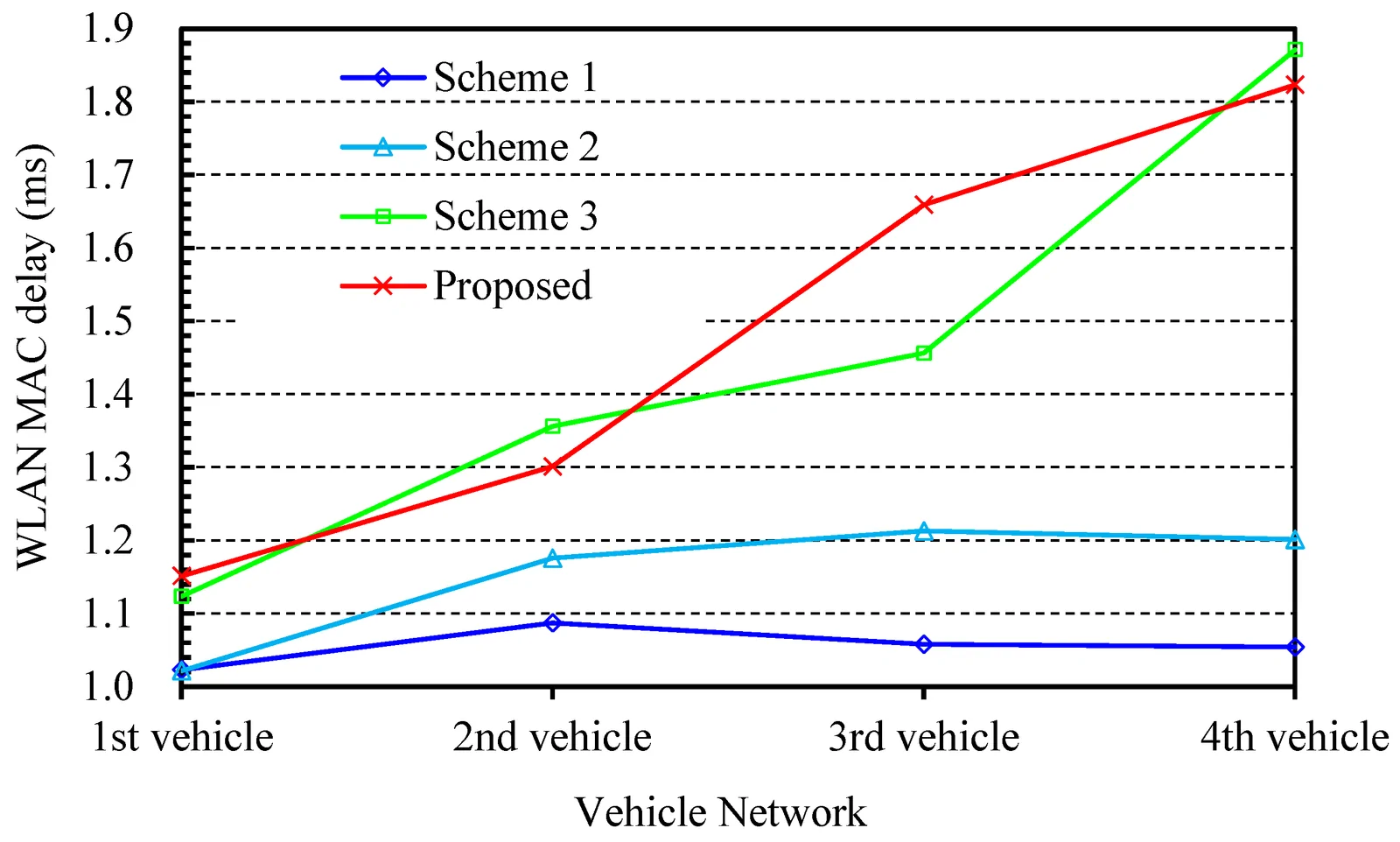
WLAN MAC delay in vehicle network.

Correspondingly, the evolutionary trajectory of the WLAN MAC delay across the terrestrial multi-hop vehicular network, as depicted in Figure 4, serves as a direct spatial extension of the traffic injection characteristics shaped by the upper UAV relay. For traditional Scheme 1 and Scheme 2, the MAC access delay across the cascading vehicular nodes remains anomalously suppressed, forming a nearly flat curve that hovers marginally between 1.0 ms and 1.2 ms up to the 4th vehicle. This low-delay plateau, however, does not indicate highly efficient spatial reuse. Rather, it is a pathological downstream manifestation of the severe starvation state propagating from the UAV layer; because the transport layer erroneously throttles the congestion window in response to random wireless errors, the terrestrial multi-hop channels remain largely idle, largely failing to sustain the data flow required for continuous vehicular communications.

On the other hand, the aggressive window expansion strategy of Scheme 3 precipitates a severe cascading collision effect on the ground. As unmanaged, bursty traffic floods the terrestrial multi-hop topology, the MAC contention accumulates non-linearly along the forwarding chain. This phenomenon culminates in a significant divergence at the network edge, where the MAC delay for Scheme 3 skyrockets to approach 1.9 ms at the 4th vehicle. Such acute delay inflation clearly indicates that the blind packet-injection strategy at the transport layer severely overwhelms the underlying medium access mechanism, severely exacerbating hidden terminal collisions and rendering the multi-hop pipeline highly unstable.

Crucially, the proposed scheme demonstrates superior cross-layer robustness and spatial fairness. While its MAC delay inherently accumulates as the hop count increases—progressing steadily from approximately 1.15 ms at the 1st vehicle to around 1.82 ms at the 4th vehicle—it successfully avoids the hazardous terminal divergence observed in Scheme 3. The proposed algorithm effectively prevents transport-layer micro-bursts from cascading into severe physical-layer collisions across multiple hops. This bounded and predictable delay growth validates that the mechanism successfully harmonizes high-throughput distribution with underlying channel contention, keeping the multi-hop transmission within a highly resilient operational envelope.

### 5.3. Queuing Delay Analysis for UAV Relay Nodes and Multi-Hop Vehicular Networks

#### 5.3.1. Queuing Delay at UAV Relay Nodes

**Figure 5:**
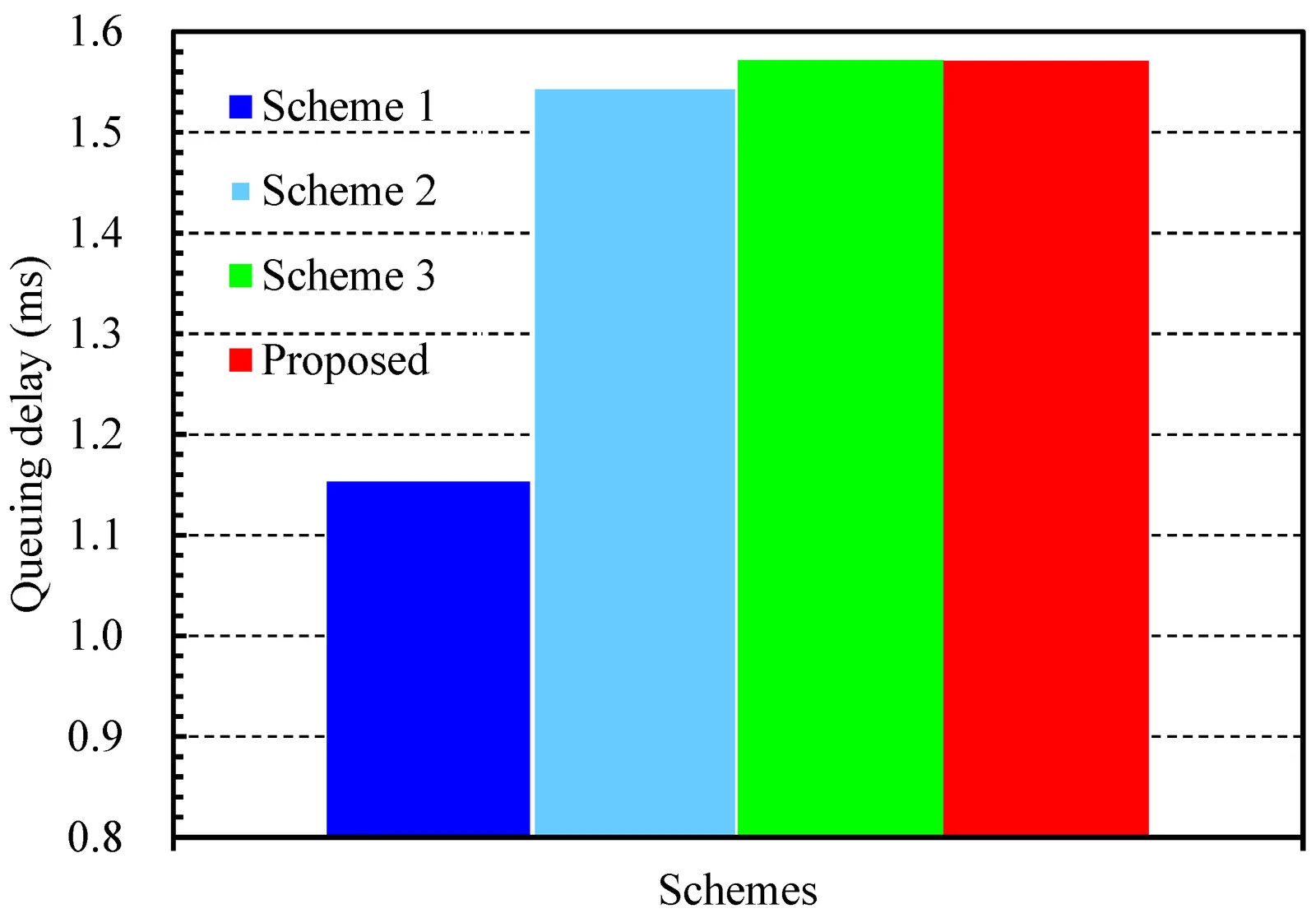
Queuing delay for UAV relay.

The queuing delay at bottleneck node buffers directly maps the throughput carrying capacity and dynamic scheduling stability of end-to-end congestion control mechanisms when confronting cascaded long delays. Simulation results reveal starkly different queuing dynamics at the relay node buffer under BER = $1\times 10^{-5}$, as shown in Figure 5.

Low-utilization idle state of Scheme 1/Scheme 2: Scheme 1’s queuing delay presents an extremely low value of 1.153 ms. This ultra-low delay occurs because Scheme 1 fails to decouple transient network congestion from random error losses, frequently triggering blind window halving and protracted backoffs. This leaves the UAV relay node’s buffer queue in a perpetual state of “starvation,” severely shrinking network throughput. Although Scheme 2 regains partial awareness via RTT variations, raising its queuing delay to 1.545 ms, its first derivative for congestion recovery remains excessively conservative, failing to fully extract potential pipe capacity.

Aggressive overcompensation and “bufferbloat” of Scheme 3: Scheme 3’s queuing delay acutely spikes to 1.571 ms. Scheme 3 relies on a pure velocity-dimension compensation function for aggressive packet injection. While elevating instantaneous pps metrics, this control loop lacks bottleneck depth feedback, causing massive data packets to indiscriminately accumulate within the relay buffer. This incurs unpredictable queuing time costs and increases the risk of “bufferbloat,” potentially inducing widespread secondary cascaded drops from buffer overflows.

Fine-grained balancing and multi-stable piecewise regulation of the Proposed Scheme: The queuing delay of the proposed scheme at the UAV relay node stabilizes at 1.570 ms. The scheme maintains a high-level relay buffer fill rate equivalent to Scheme 3 to guarantee maximized high-pps throughput, while concurrently achieving refined, adaptive stabilization of the queuing depth. Its mathematical core originates from the queue-aware piecewise control law. When the virtual queue backlog estimator determines link bandwidth is abundant (State A), it implements an active bandwidth probing update equation (Formula (8)). Once the virtual backlog breaches the decision threshold (State B), it immediately attenuates the single-step update stride (Formula (9)). By implicitly introducing an acknowledgment counter to artificially suppress the first derivative of window growth, it achieves an optimal control trade-off between maximizing throughput and preventing queue overload.

#### 5.3.2. Queuing Delay at Terrestrial Vehicular Receiving Nodes

**Figure 6:**
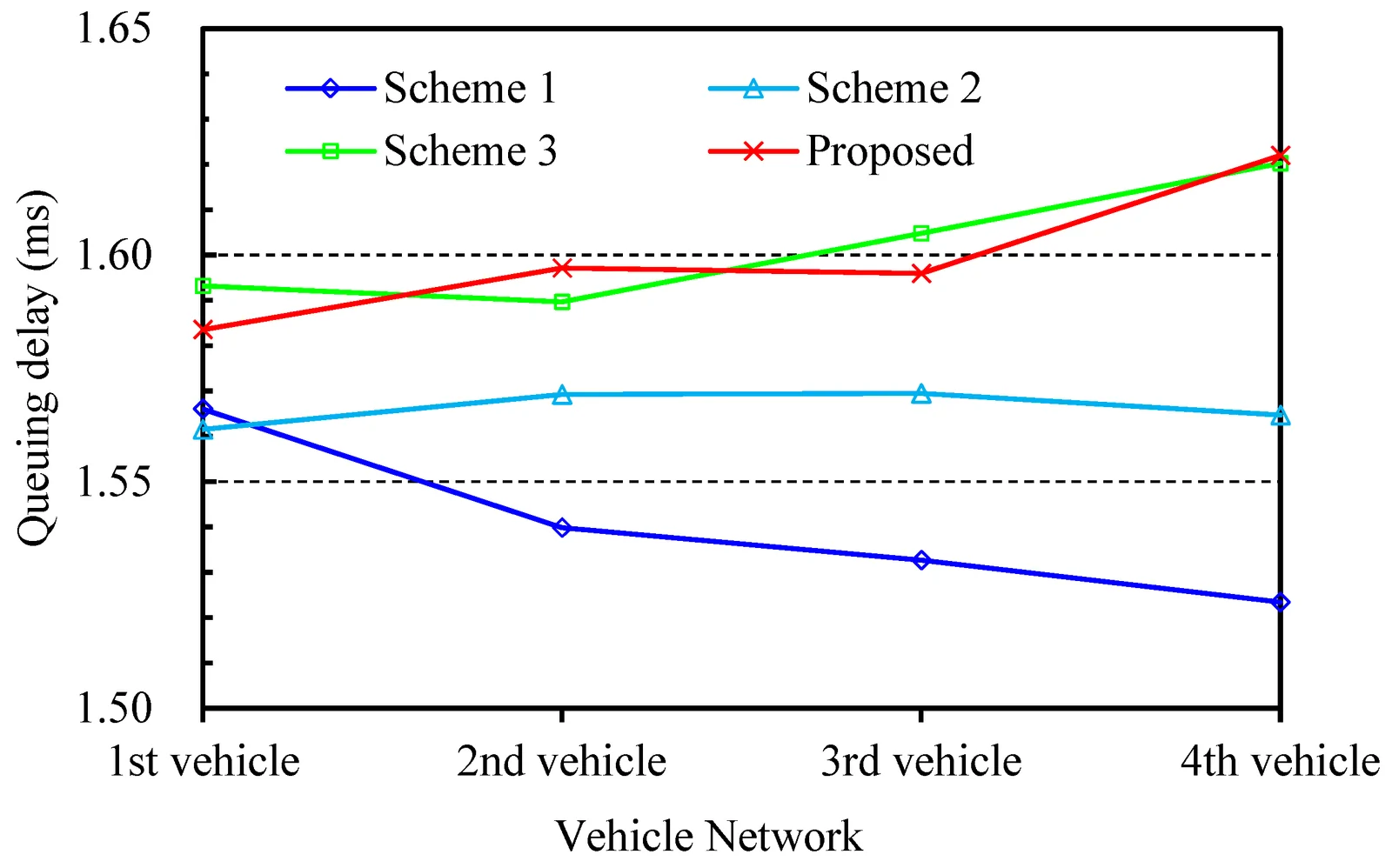
Queuing delay in vehicle network.

Figure 6 traces the evolutionary trajectory of receiver queuing delays along the multi-hop chain network extension to evaluate spatial fairness and divergence-suppression capabilities.

Conventional Scheme 1, constrained by the cascading amplification of the loss-triggering causal chain due to accumulated multi-hop errors, suffers a gradual destabilization, dropping from 1.566 ms at the first vehicle to 1.523 ms at the fourth. Its remote edge nodes fail to establish a coherent streaming data reception stack. Correspondingly, Scheme 3 exhibits a hazardous divergence trend in queuing delay, leaping monotonically from 1.593 ms at the first vehicle to 1.620 ms at the fourth. This exposes the detrimental multi-hop channel hoarding effect imposed by blindly inflating the sending window; severe MAC-layer retransmissions and collisions prevent the edge nodes’ queues from emptying promptly.

The proposed optimization algorithm manifests highly robust convergence characteristics, recording queuing delays of 1.583 ms, 1.597 ms, 1.595 ms, and 1.621 ms across nodes 1 through 4. The algorithm ensures network edge nodes maintain a rational dynamic queuing depth to support high-concurrency multi-hop distribution. By adaptively sensing scale factor variations across vehicular links at different spatial scales, it successfully severs the delay divergence chain, achieving superior contract fairness and dynamic scheduling stability across the global topology.

### 5.4. Closed-Loop Convergence Deconstruction of Cross-Layer Delay Misalignment Gap

By comprehensively comparing the WLAN MAC layer delay with the bottleneck node queuing delay, a core scientific phenomenon regarding cross-layer design synergy is revealed: In conventional schemes, there exists a profound macroscopic misalignment and asymmetric cross-layer gap between the WLAN MAC delay fluctuation range and the queuing delay distribution, whereas the proposed scheme successfully compresses this delay gap, achieving deep closed-loop assimilation between transport-layer injection behaviors and link-layer channel access characteristics (see Table 1).

Mechanistically, because Scheme 1 and Scheme 2 suffer frequent, unnecessary window collapses upon encountering random errors, their extremely low MAC delays represent a pathological mismatch acquired at the expense of chronic queue starvation and a total collapse in throughput. Conversely, while Scheme 3 elevates the queuing delay to a saturated state of 1.571 ms, its lack of fine-grained elastic decoupling concerning the underlying network queue backlog causes the bursty traffic handed down to exhibit high disorder. This unsmoothed packet injection directly exceeds the data link layer’s carrying limit, triggering severe BEB backoff collisions at the MAC layer, forcing the MAC delay to experience runaway inflation to 1.091 ms.

In contrast, under the requirement of operating the relay node queue at a high load of 1.570 ms to guarantee maximum theoretical throughput, the corresponding WLAN MAC delay of the proposed scheme undergoes a reverse micro-adjustment, flattening to 1.085 ms. This enables the relative decoupled gap between queuing delay and MAC access delay to be finely constrained within an optimal equilibrium domain of 0.485 ms. It should be noted that a smaller delay gap ΔT is not strictly better. For instance, Scheme 1’s lowest gap is a pathological result of queue starvation. The proposed scheme dynamically constrains the gap within an optimal equilibrium domain of 0.485 ms, ensuring deep closed-loop assimilation.

This convergence phenomenon strongly substantiates that the piecewise additive increase control law driven jointly by the continuous-time spatiotemporal scale normalization theory and the virtual queue backlog indicator adaptively derives an implicit cross-layer-aware pacing effect within the end-to-end control loop. By proactively probing on demand when the bottleneck is unsaturated and instantaneously suppressing the first derivative of window evolution under critical congestion states, the algorithm flexibly aligns the packet injection behavior of the transport protocol with the microscopic medium access rate of the underlying physical channel. This refined adaptive rate regulation fundamentally circumvents the intense induced channel contention at the underlying MAC layer caused by chaotic micro-bursts and effectively mitigates the pathological delay misalignment gap across the protocol stack.

## 6. Conclusions

This paper investigated the reliable transmission optimization problem in UAV-relayed space–air–ground integrated vehicular networks (SAGVNs), where the coexistence of long-delay satellite links, highly dynamic UAV-assisted relay paths, and multi-hop vehicular communications poses significant challenges to conventional transport control mechanisms. Unlike terrestrial networks with relatively stable link characteristics, the heterogeneous SAGVN environment exhibits severe latency variations and frequent non-congestion packet losses triggered by channel fading, topology changes, and mobility-induced link disruptions. These characteristics make it difficult for existing loss-based and delay-based congestion control schemes to maintain stable and efficient end-to-end transmission performance. To address these issues, a reliable transmission optimization scheme was developed by jointly considering the dynamic characteristics of heterogeneous links and the evolutionary behavior of transport-layer states. First, a virtual queue backlog estimation method was introduced to characterize the actual congestion state at bottleneck relay nodes, enabling the accurate differentiation of congestion-induced packet losses from random transmission errors. Furthermore, a continuous-time spatiotemporal normalization model was established to mitigate the unfairness induced by heterogeneous round-trip times (RTTs). Building upon these models, a queue-aware nonlinear window adjustment strategy was designed to adaptively regulate transmission rates according to real-time network states, thereby improving bandwidth utilization while preventing excessive buffer accumulation. Extensive simulation results under high-latency and high-error-rate conditions demonstrate that the proposed scheme achieves superior transmission performance compared with baseline protocols, including TCP Reno, TCP Veno, and TCP Hybla. In particular, the proposed mechanism significantly enhances link throughput while maintaining stable queue dynamics at both UAV relay nodes and vehicular terminals. These results verify that coupling delay-scale normalization with congestion state awareness effectively overcomes the performance limitations of traditional transport protocols in UAV-assisted heterogeneous networks. Although this work focuses on transport-layer optimization for UAV-relayed SAGVNs, several open issues warrant further investigation. Future research will explore joint optimization across multiple protocol layers, including adaptive routing, resource allocation, and intelligent relay selection. Additionally, incorporating learning-based prediction mechanisms to handle dynamic topology evolution and channel variations could further enhance the adaptability of reliable transmission schemes in large-scale, highly dynamic space–air–ground integrated networks. Furthermore, while this study establishes a strong foundation against classic baseline protocols, future work will extend the evaluation to modern congestion control algorithms, including BBR and CUBIC, by migrating to advanced simulation platforms or Hardware-in-the-Loop (HiL) testbeds. We also plan to integrate Gilbert–Elliott burst-loss channel models to more accurately replicate realistic fading environments in lower-altitude airspaces.

## Figures and Tables

**Table 1:** Quantitatively extracts this physical parameter of delay gap ($\Delta T=T_{queue}-T_{mac}$).

Scheme	UAV Relay Queuing Delay Tqueue (ms)	WLAN MAC Medium Access Delay Tmac (ms)	Cross-Layer Delay Misalignment Gap ΔT (ms)
Scheme 1	1.153	0.898	0.255
Scheme 2	1.545	0.951	0.594
Scheme 3	1.571	1.091	0.48
Proposed Scheme	1.57	1.085	0.485

## Data Availability

The original contributions presented in this study are included in the article. Further inquiries can be directed to the corresponding authors.
